# Diabetes mellitus and obesity among South Asians with ischemic stroke across three countries

**DOI:** 10.1177/17474930231203149

**Published:** 2023-09-29

**Authors:** Gie Ken-Dror, Intisar Ajami, Thang S Han, Taylor Aurelius, Ankita Maheshwari, Hassan Al Hail, Dirk Deleu, Sapna D Sharma, Sageet Amlani, Gunaratnam Gunathilagan, David L Cohen, Chakravarthi Rajkumar, Stuart Maguire, Sissi Ispoglou, Ibrahim Balogun, Anthea Parry, Lakshmanan Sekaran, Hafiz Syed, Enas Lawrence, Ravneeta Singh, Ahamad Hassan, Chris Wharton, Khalid Javaid, Neetish Goorah, Peter Carr, Eman Abdus Sami, Musab Ali, Hassan Al Hussein, Hassan Osman Abuzaid, Khalid Sharif, Shri Ram Sharma, PN Sylaja, Fahmi Yousef Khan, Kameshwar Prasad, Pankaj Sharma

**Affiliations:** 1 Institute of Cardiovascular Research Royal Holloway, University of London (ICR2UL), London, UK; 2 Department of Endocrinology, Ashford and St Peter’s Hospitals NHS Foundation Trust, Surrey, UK; 3 Departments of Neurology, All India Institute of Medical Sciences, New Delhi & Rajendra Institute of Medical Sciences, Ranchi, India; 4 Hamad Medical Corporation, Doha, Qatar; 5 Department of Neurology, Neuroscience Institute, Hamad Medical Corporation, Doha, Qatar; 6 BARTS and the London NHS Trust, Royal London Hospital, London, UK; 7 Queen Elizabeth the Queen Mother Hospital, Kent, UK; 8 Northwick Park Hospital, London, UK; 9 Brighton and Sussex University Hospitals NHS Trust & Brighton and Sussex Medical School, University of Sussex, Sussex, UK; 10 Bradford Teaching Hospital, West Yorkshire, UK; 11 Birmingham City Hospital, West Midlands, UK; 12 William Harvey Hospital, Kent, UK; 13 Hillingdon Hospital, London, UK; 14 Luton and Dunstable Hospital, Bedfordshire, UK; 15 Newham University Hospital, London, UK; 16 Croydon University Hospital, London, UK; 17 West Middlesex University, London, UK; 18 Leeds General Infirmary, West Yorkshire, UK; 19 New Cross Hospital, West Midlands, UK; 20 Walsall Manor Hospital, West Midlands, UK; 21 Queen’s Park Hospital Royal Blackburn, Lancashire, UK; 22 Birmingham Heartlands Hospital, West Midlands, UK; 23 Airedale General Hospital, West Yorkshire, UK; 24 North Eastern Indira Gandhi Regional Institute for Health and Medical Sciences, Shillong, Meghalaya, India; 25 Department of Neurology, Sree Chitra Tirunal Institute for Medical Sciences and Technology, Trivandrum, India; 26 Ashford and St Peter’s NHS Foundation Trust, Surrey, UK; 27 Department of Clinical Neuroscience, Imperial College Healthcare NHS Trust, London, UK

**Keywords:** Stroke, diabetes, obesity, ethnicity

## Abstract

**Background:**

Diabetes mellitus and central obesity are more common among South Asian populations than among White British people. This study explores the differences in diabetes and obesity in South Asians with stroke living in the United Kingdom, India, and Qatar compared with White British stroke patients.

**Methods:**

The study included the UK, Indian, and Qatari arms of the ongoing large Bio-Repository of DNA in Stroke (BRAINS) international prospective hospital-based study for South Asian stroke. BRAINS includes 4580 South Asian and White British recruits from UK, Indian, and Qatar sites with first-ever ischemic stroke.

**Results:**

The study population comprises 1751 White British (WB) UK residents, 1165 British South Asians (BSA), 1096 South Asians in India (ISA), and 568 South Asians in Qatar (QSA). ISA, BSA, and QSA South Asians suffered from higher prevalence of diabetes compared with WB by 14.5% (ISA: 95% confidence interval (CI) = 18.6–33.0, *p* < 0.001), 31.7% (BSA: 95% CI = 35.1–50.2, *p* < 0.001), and 32.7% (QSA: 95% CI = 28.1–37.3, *p* < 0.001), respectively. Although WB had the highest prevalence of body mass index (BMI) above 27 kg/m^2^ compared with South Asian patients (37% vs 21%, *p* < 0.001), South Asian patients had a higher waist circumference than WB (94.8 cm vs 90.8 cm, *p* < 0.001). Adjusting for traditional stroke risk factors, ISA, BSA, and QSA continued to display an increased risk of diabetes compared with WB by 3.28 (95% CI: 2.53–4.25, *p* < 0.001), 3.61 (95% CI: 2.90–4.51, *p* < 0.001), and 5.24 (95% CI: 3.93–7.00, *p* < 0.001), respectively.

**Conclusion:**

South Asian ischemic stroke patients living in Britain and Qatar have a near 3.5-fold risk of diabetes compared with White British stroke patients. Their body composition may partly help explain that increased risk. These findings have important implications for public health policymakers in nations with large South Asian populations.

## Introduction

One in five strokes in Britain are caused by type 2 diabetes which doubles the risk of stroke within the first 5 years of diagnosis.^1^ In the United Kingdom, 3.6 million persons, around 5% of the population, have been diagnosed with diabetes, with diabetes mellitus type 2 accounting for around 90% of cases,^2^ while an additional million people are thought to suffer from undiagnosed diabetes. Diabetes and central obesity are twice as common among South Asians from India, Pakistan, and Bangladesh living in the United Kingdom than among White British people^3^ often striking them earlier in life.^4^

South Asian populations have a 15–27% greater prevalence of diabetes mellitus than White British populations.^3^ Gender does not appear to influence differences between these two ethnic groups.^5^ Moreover, this elevated prevalence is still present in individuals with first-onset stroke.^6^ Diabetes is 80 times more likely to occur in obese adults than in healthy individuals with body mass indices (BMIs) under 22.^7^ South Asians generally have a lower prevalence of overweight/obesity than the White British population, men having the largest difference in ethnic prevalence (16.3%) and women having the smallest difference (1%), even though there is little, if any, data on prevalence among those who have ischemic stroke.^5^ South Asian infants born in the United Kingdom had lower birth weights, which may have a hereditary basis.^8^ It is important to note the differences in fat distribution that exist throughout the South Asian community, particularly in view of the effect that these measures have for the prevalence of obesity. South Asians have greater accumulation of visceral and subcutaneous fat around the abdomen as well as increased skinfold thickness compared with their White British counterparts.^9^ It has already been determined that increased central adiposity in South Asians increases ischemic stroke risk factors, such as elevated C-reactive protein (CRP) concentration.^10^ Furthermore, it has been hypothesized that South Asian’s atherogenic lipid profile is a result of rising central adiposity.^11^

Migration and cultural norms around nutrition and exercise have been presumed to significantly compound the increased prevalence of diabetes mellitus among ethnic South Asians. However, the assumption that South Asians who immigrate to other nations have a higher incidence of diabetes, obesity, and visceral fat location compared with local White populations has not been robustly tested using large and comparative international datasets. Demonstrating differences in disease occurrence following migration is likely to have important public health implications for nations with sizable South Asian populations.

We aimed to investigate the disparities in diabetes mellitus and obesity between South Asians in the United Kingdom, India, and Qatar with a White British population among patients who have had first-time an ischemic stroke using one of the largest such datasets currently available.

## Materials and methods

### Data source

We used the UK, Indian, and Qatar arms of the ongoing large prospective international Bio-Repository of DNA in Stroke (BRAINS) study, the details of which have been previously published^12,13^ but are described in detail in the Supplementary material. However, briefly, ischemic stroke patients of South Asian descent were recruited from 21 sites in the United Kingdom, 2 in India (North and South), and 1 in Qatar. Detailed phenotypic and demographic information was documented.^12,13^ Stroke in all patients was confirmed by computed tomography (CT) and/or magnetic resonance imaging (MRI) of the brain. All patients were reviewed by a stroke or neurology physician.

### Statistical analysis

Descriptive statistics summarized data using mean with standard deviation (SD) or median with interquartile range (IQR) for continuous variables, and proportion for categorical variables. For single-factor analysis, chi-square (or Fisher exact test, where appropriate) was used for categorical variables, and independent *t*-test (or Mann–Whitney *U* test, where appropriate) for continuous variables. Univariate and multivariate logistic regression estimated the associations of covariates (age, sex, central obesity, smoking history, alcohol consumption, hypertension, hypercholesterolemia, and cardiovascular diseases) with diabetes mellitus status. The reliability (goodness-of-fit) of each model was quantified using the Hosmer and Lemeshow test. Models evaluated using Akaike’s information criterion (AIC) and the likelihood ratio chi-square test. Rather than applying a correction for multiple testing at global significance level, for individual tests of association defined statistical significance was <0.01.

## Results

The study population was 4580 individuals identified with first-time ischemic stroke comprising 1751 (men: 980, women: 771) White British (WB), 1165 (men: 752, women: 413) British South Asians (BSA), 1096 (men: 739, women: 359) South Asians in India (ISA), and 568 (men: 543, women: 25) South Asians in Qatar (QSA) patients. The mean age was 62.2 ± 16.0 and 65.8% (95% confidence interval (CI): 64.4–67.2) of the sample were male. The prevalence among first-time ischemic stroke patients of diabetes was 34.1% (95% CI: 32.8–35.5), hypertension 67.8% (95% CI: 66.4–69.1), hypercholesterolemia 39.1% (95% CI: 37.6–40.6), smoking history 18.6% (95% CI: 17.4–19.7), and alcohol consumption 36.0% (95% CI: 34.6–37.5).

Demographic and clinical characteristics by diabetes status and ethnicity are presented in Table 1. Diabetic patients compared with non-diabetic patients suffered from higher adiposity index in all ethnic groups. Diabetic patients had a higher average BMI and waist circumference value than non-diabetic patients by 0.94 kg/m^2^ (95% CI: 0.59–1.28, *p* < 0.001) and 6.7 cm (95% CI: 5.32–8.09, *p* < 0.001), respectively. In addition, diabetic patients had higher prevalence of central obesity by 5.6% (95% CI: 2.6–8.6, *p* < 0.001) compared with non-diabetic patients. Diabetic patients had an increased prevalence of diabetes family history by 31.2% (95% CI: 27.9–34.5, *p* < 0.001), hypertension by 23.6% (95% CI: 21.0–26.2, *p* < 0.001), hypercholesterolemia by 22.0% (95% CI: 18.9–25.1, *p* < 0.001), and cardiovascular disease by 11.7% (95% CI: 9.1–14.3, *p* < 0.001) compared with non-diabetic patients. No significant difference was observed in gender between diabetes and non-diabetes patients.

**Table 1.** Population characteristics and comorbidities by ethnicity and diabetes status.

	WB			BSA			ISA			QSA		
	Diabetes (*n* = 325) Mean (SD)	Non-diabetes (*n* = 1426) Mean (SD)	*p*-value	Diabetes (*n* = 585) Mean (SD)	Non-diabetes (*n* = 580) Mean (SD)	*p*-value	Diabetes (*n* = 362) Mean (SD)	Non-diabetes (*n* = 734) Mean (SD)	*p*-value	Diabetes (*n* = 291) Mean (SD)	Non-diabetes (*n* = 277) Mean (SD)	*p*-value
Average age of onset (years, SD)	71.1 (11.6)	71.4 (13.8)	0.65	65.4 (12.6)	62.4 (17.2)	<0.001	56.3 (9.9)	49.9 (14.2)	<0.001	50.9 (9.7)	50.9 (11.0)	0.97
Men, *n* (%)	197 (60.6)	783 (54.9)	0.071	375 (64.1)	377 (65.0)	0.80	256 (70.7)	483 (65.8)	0.12	282 (96.9)	261 (94.2)	0.18
Smoking history, *n* (%)	842 (13.9)	193 (15.2)	0.67	96 (16.5)	87 (15.1)	0.77	63 (17.5)	128 (17.5)	0.31	95 (32.6)	114 (41.2)	<0.001
Alcohol consumption, *n* (%)	93 (33.1)	583 (49.1)	<0.001	115 (20.6)	139 (25.0)	0.089	149 (41.4)	309 (42.6)	0.76	60 (20.6)	77 (27.8)	0.057
Adiposity index												
BMI (kg/m^2^)	29.2 (6.1)	26.7 (6.0)	<0.001	26.7 (5.0)	25.9 (5.1)	0.007	24.7 (3.7)	23.5 (4.4)	<0.001	26.4 (4.1)	26.2 (3.9)	0.58
Waist circumference (cm)	97.4 (20.6)	89.2 (16.2)	<0.001	100.4 (16.7)	93.8 (14.2)	<0.001	101.7 (22.0)	91.9 (18.0)	<0.001	92.2 (11.5)	91.0 (11.3)	0.22
Central obesity, *n* (%)	116 (51.6)	336 (34.1)	<0.001	193 (37.2)	138 (27.5)	<0.001	52 (16.0)	56 (9.0)	0.002	41 (14.1)	41 (14.8)	0.90
Comorbidities, *n* (%)												
Diabetes family history	107 (46.1)	194 (18.7)	<0.001	318 (71.5)	232 (58.6)	<0.001	161 (45.6)	142 (19.7)	<0.001	129 (56.6)	38 (18.2)	<0.001
Hypertension	257 (79.3)	834 (58.6)	<0.001	510 (88.1)	338 (58.6)	<0.001	296 (82.7)	423 (59.1)	<0.001	229 (79.0)	192 (69.3)	0.011
Hypercholesterolemia	165 (50.8)	419 (29.8)	<0.001	373 (66.1)	214 (37.8)	<0.001	149 (45.0)	200 (31.6)	<0.001	121 (41.9)	75 (27.4)	<0.001
Cardiovascular diseases	81 (27.1)	224 (17.6)	<0.001	218 (38.4)	110 (19.4)	<0.001	63 (17.5)	79 (10.9)	0.003	39 (13.5)	8 (2.9)	<0.001

n: sample size; WB: White British individuals living in the United Kingdom; BSA: British South Asians living in the United Kingdom; ISA: South Asians living in India; QSA: South Asians living in Qatar; SD: standard deviation; BMI: body mass index; MI: myocardial infarction.

Central obesity was defined by waist circumference (men: >102 cm, women: >88 cm) or body mass index (⩾27 kg/m^2^); cardiovascular diseases was defined by the presence of ischemic heart disease, angina, or MI.

**Figure 1.**
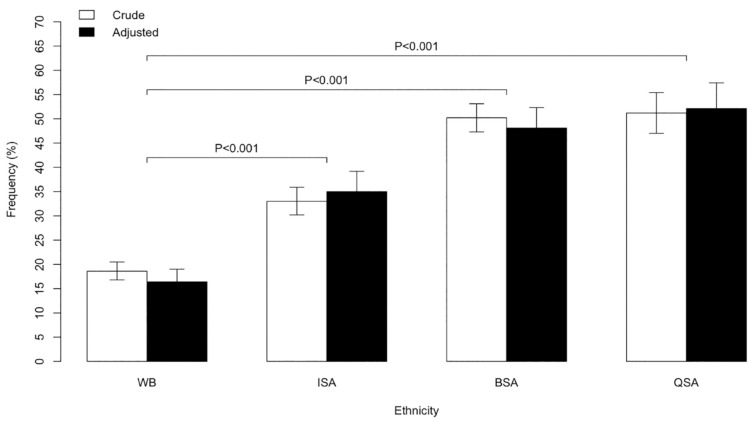
Prevalence of diabetes among WB, ISA, BSA, and QSA. WB: White British individuals living in the United Kingdom; BSA: British South Asians living in the United Kingdom; ISA: South Asians living in India; QSA: South Asians living in Qatar. Adjusted for age and sex.

The prevalence of diabetes among WB, ISA, BSA, and QSA is presented in Figure 1. South Asian patients had a higher prevalence of diabetes compared with White British patients among first-time ischemic stroke patients. ISA had higher prevalence of diabetes compared with WB by 14.5% (95% CI: 18.6–33.0, *p* < 0.001), BSA by 31.7% (95% CI: 35.1–50.2, *p* < 0.001), and QSA by 32.7% (95% CI: 28.1–37.3, *p* < 0.001). Migrated South Asian patients BSA and QSA patients suffer from greater prevalence of diabetes compared with ISA patients by 17.2% (95% CI: 13.0–21.2, *p* < 0.001) and 18.2% (95% CI: 13.1–23.3, *p* < 0.001), respectively.

**Figure 2.**
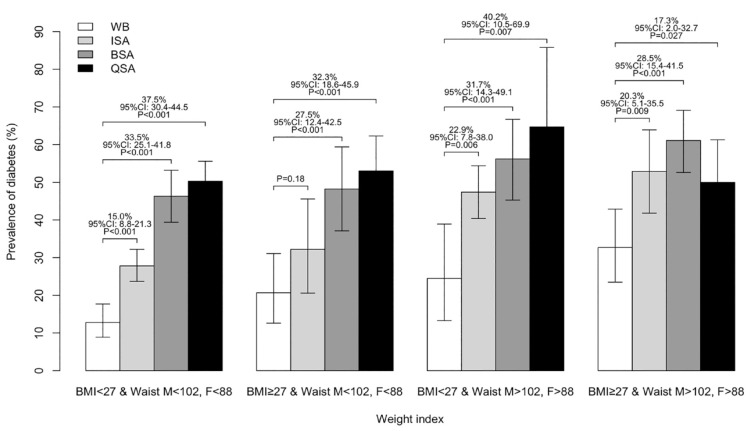
Prevalence of diabetes among BMI above or below 27 kg/m^2^ and waist circumference above or below 102 cm in male and 88 cm in female. WB: White British individuals living in the United Kingdom; BSA: British South Asians living in the United Kingdom; ISA: South Asians living in India; QSA: South Asians living in Qatar.

The prevalence of diabetes by BMI above or below 27 kg/m^2^ and waist circumference above or below 102 cm in male and 88 cm in female are presented in Figure 2. The BSA and QSA had the highest prevalence of diabetes in all four combinations of BMI and waist circumference. This was followed by ISA who had the third highest prevalence.

The association between diabetes status and ethnic group among ischemic stroke patients was evaluated using logistic regression (Table 2). Univariate analysis showed that south Asian ethnicity (ISA, BSA, QSA) was associated with diabetes status. The odds ratio (OR) for diabetes presence was 3.41 (95% CI: 2.97–3.94, *p* < 0.001) for South Asians (ISA, BSA, QSA) versus WB. Multivariate logistic regression analysis modeling including traditional stroke risk factors (age, sex, central obesity, smoking history, alcohol consumption, hypertension, hypercholesterolemia, and cardiovascular diseases) showed that ethnicity independently associated with diabetes (Table 2). Multivariate adjusted OR for South Asian ethnicity (ISA, BSA, QSA) compared with WB was 3.68 (95% CI: 2.99–4.53, *p* < 0.001).

**Table 2.** Multivariate logistic regression analysis of diabetes status.

	Model 1 OR (95% CI), *p*-value	Model 2 OR (95% CI), *p*-value	Model 3 OR (95% CI), *p*-value
WB	Reference group	Reference group	Reference group
ISA	2.16 (1.82–2.58), <0.001	2.81 (2.30–3.43), <0.001	3.28 (2.53–4.25), <0.001
BSA	4.43 (3.75–5.23), <0.001	4.91 (4.13–5.84), <0.001	3.61 (2.90–4.51), <0.001
QSA	4.61 (3.76–5.66), <0.001	5.79 (4.58–7.34), <0.001	5.24 (3.93–7.00), <0.001

OR: odds ratio; CI: confidence interval; WB: White British individuals living in the United Kingdom; ISA: South Asians living in India; BSA: British South Asians living in the United Kingdom; QSA: South Asians living in Qatar.

Model 1: Unadjusted.

Model 2: Adjusted for age and sex.

Model 3: Adjusted for age, sex, central obesity, smoking history, alcohol consumption, hypertension, hypercholesterolemia, and cardiovascular diseases.

## Discussion

South Asian ISA, BSA, and QSA ischemic stroke patients compared with their WB stroke counterparts suffer from higher prevalence of diabetes by 15%, 32%, and 33%, respectively, following adjustment for traditional risk factors (age, sex, central obesity, smoking history, alcohol consumption, hypertension, hypercholesterolemia, and cardiovascular diseases). Moreover, migrated South Asians present with a greater prevalence of diabetes compared with those who remain in the subcontinent. These results reflect differences in adiposity index, BMI, waist circumference, and central obesity among South Asian patients compared with WB and ISA, likely highlighting differences in lifestyle and environmental factors such as diet and exercise.

The body composition of the South Asian patients may help to explain the increased risk of developing diabetes compared with the WB patients. A high visceral fat percentage has been highly correlated with having a greater waist circumference. South Asians tend to have higher amounts of abdominal adipose tissue, including both subcutaneous and visceral fat than their Caucasian counterparts at similar BMI values, although no differences were found at very high values of BMI.^14^ Observational studies have shown that excessive amounts of visceral adipose tissue can increase the risk of developing diabetes due to it being the primary causal factor of insulin resistance.^15^

Previous studies indicated that South Asian populations are more likely to have abdominal obesity and more body fat at a given BMI value than Caucasians.^16^ Due to this, they are at an increased risk of developing diabetes and other comorbidities, including hypertension, stroke, cardiovascular disease, and hypercholesterolemia.^17^ Several studies have demonstrated that South Asians are at risk of developing obesity-related comorbidities at lower BMI levels or smaller waist circumference measures.^18^

BMI does not distinguish between muscle and fat.^16^ A person with a high proportion of muscle may be considered overweight or even obese since muscle weighs more than fat. Moreover, BMI ignores the distribution of fat, despite that being important for predicting health outcomes.^16^ The risk of diabetes and heart disease is higher in people with “apple” shapes who carry fat around their middle but may be quite thin elsewhere.^16^ Individuals with this body type could be considered healthy according to the BMI calculation.^16^ “Pear” shapes obesity formally classified as “overweight,” despite the fact that fat stored around the hips, bottom, and thighs is more secure. Asian-descent individuals have higher weight-related disease risks at lower BMIs and more likely to develop diabetes and heart disease, due to central fat.^16^ This requires separate standards for various ethnic groups. As waist circumference is a stronger predictor of type 2 diabetes risk, regardless of BMI,^19^ it presents a powerful argument for a wider adoption of more ethnic-specific criteria.^20^

Release of adipokines and their associated metabolic consequences may partly explain why South Asians have a higher risk of diabetes due to an excessive build-up of adipose tissue. A secreted adipokine known as adiponectin is thought to play a role in the modulation of glucose and lipid metabolism in insulin-sensitive tissues with lower levels of blood adiponectin implicated in the pathogenesis of insulin resistance and diabetes.^21^ Unlike their Caucasian counterparts, South Asian men have been found to have lower levels of adiponectin despite their body fat content and body fat distribution.^22^ A correlation between lower levels of adiponectin and insulin has been previously identified, as well as the identification of lower levels of adiponectin in South Asians with diabetes.^21,22^ Adipocytes also produce leptin which is known to have insulin-sensitizing effects as well as causing a reduction in a person’s appetite.^23^ Obese individuals tend to develop hyperleptinemia which causes them to become leptin resistant which contributes to insulin resistance as it suppresses insulin action.^23^ South Asians have been found to have higher levels of leptin in the system than their Caucasian counterparts, despite their body fat distribution or body composition.^21^ Leptin levels have been found to be higher in South Asians with diabetes than those with only an impaired glucose tolerance or normal glucose tolerance.^22^

The higher prevalence of diabetes in South Asians in the United Kingdom and Qatar compared with Indian South Asians highlights the possible changes in lifestyle and environmental factors, including nutrition and exercise that may occur when people migrate. The traditional South Asian carbohydrates heavy diet of rice and bread is better suited to a physically demanding rural environment.^24^ Yet South Asians in the United Kingdom continue to eat a high-carbohydrate diet and engage in less exercise than the WB population.^25^ The World Health Organization (WHO) has recommended special standards for determining obesity in South Asians due to a tendency toward visceral adiposity in the truncal region.^20^ Another potential element in the variation in diabetes risk is most likely the impact of migration on environmental and lifestyle factors. In migration studies, access to healthcare is a crucial component of diabetes prevention that is frequently ignored. Despite having access to healthcare facilities that can help prevent disease, BSA have demonstrably limited awareness of the risk factors that contribute to disease. South Asian patients in the United Kingdom and Qatar may be less informed of the typical complications linked to diabetes mellitus, the significance of screening clinics, and the necessity of consulting chiropodists.^26^ In addition, sociocultural and religious factors might compound this diminished awareness by creating false notions of social stigma and failure to self-care.^27^

Diabetes mellitus may be prevented in South Asian communities using programs that promote health-protective lifestyle changes and comprise multi-component interventions that encompass parts of health promotion and behavioral change that are passive (lecture-based), active (activity-based), and individualized (such as targeted counseling).^28^ These programs should focus on cultural values related to lifestyle aspects such as food, which reduces carbohydrate intake, and exercise, which raising aerobic exercise at a moderate intensity.

## Limitations

As with all studies, a number of limitations need to be noted. The BRAINS project has been ongoing for several years and in that time risk factor thresholds and management approaches have evolved. However, those changes would have led to a non-differential ethnic bias and are unlikely to have an impact on the significance of the findings in this study. The prevalence of comorbidities is recorded at the time of the event. Most of the prevalence data was gathered from treatment regimens documented on the patient’s health record even though we are unable to comment on how long these comorbidities were prevalent before the diabetic event. The large sample size (*n* = 4580) accurately reflects the current influence of these risk factors on diabetes in the South Asian population, even though this may result in an underestimation of the real effect size on the diabetes event. Grandparent origin defined ethnicity. Although this was self-reported, prior research has shown that this methodology is accurate.^29^ Due to the lack of socioeconomic data collection, we are unable to determine how socioeconomic status may affect morbidity and mortality or the occurrence of developing diabetes. Using the larger BRAINS dataset which includes both ischemic and hemorrhagic events, we evaluated how well subjects were represented in our study. We discovered similar stroke subtype prevalence in all four of our study’s groups.^30^ In addition, because long-term follow-up was not conducted, we are unable to report on specific migratory effects and how they develop. The recruitment sites in the United Kingdom, India, and Qatar were selected to provide a representative sample. Twenty-one hospital locations with a strong South Asian population were identified in the United Kingdom. All India Institute of Medical Sciences and Sree Chitra Tirunal Institute for Medical Sciences and Technology were chosen as the two hospital locations since they are situated in the north and south of the nation, respectively. Different cultures have different responses and access to seeking emergency healthcare support services. However, all our recruitment sites across three countries provide free access to medical services, making it likely to draw people from a wide range of socioeconomic backgrounds. Nevertheless, this is a hospital-based study and is dependent on recruitment for patients attending hospital.

## Conclusion

South Asian ischemic stroke patients in India (ISA), Britain (BSA), and Qatar (ISQ) have higher prevalence of diabetes mellitus compared with White British stroke patients, with migrants being most affected. Body composition of South Asian patients may help to explain the increased risk of developing diabetes. These findings have important implications for public health policymakers in nations with large migrant South Asian populations.

## Supplemental Material

sj-docx-1-wso-10.1177_17474930231203149 – Supplemental material for Diabetes mellitus and obesity among South Asians with ischemic stroke across three countries Click here for additional data file. Supplemental material, sj-docx-1-wso-10.1177_17474930231203149 for Diabetes mellitus and obesity among South Asians with ischemic stroke across three countries by Gie Ken-Dror, Intisar Ajami, Thang S Han, Taylor Aurelius, Ankita Maheshwari, Hassan Al Hail, Dirk Deleu, Sapna D Sharma, Sageet Amlani, Gunaratnam Gunathilagan, David L Cohen, Chakravarthi Rajkumar, Stuart Maguire, Sissi Ispoglou, Ibrahim Balogun, Anthea Parry, Lakshmanan Sekaran, Hafiz Syed, Enas Lawrence, Ravneeta Singh, Ahamad Hassan, Chris Wharton, Khalid Javaid, Neetish Goorah, Peter Carr, Eman Abdus Sami, Musab Ali, Hassan Al Hussein, Hassan Osman Abuzaid, Khalid Sharif, Shri Ram Sharma, PN Sylaja, Fahmi Yousef Khan, Kameshwar Prasad and Pankaj Sharma in International Journal of Stroke
