# Supplementary material for: Diabetes mellitus and obesity among South Asians with ischemic stroke across three countries
Source: Int J Stroke. 2023 Sep 29;19(2):235–43. doi: 10.1177/17474930231203149 (PMC10811966; doi:10.1177/17474930231203149)
Supplement: sj-docx-1-wso-10.1177_17474930231203149 – Supplemental material for Diabetes mellitus and obesity among South Asians with ischemic stroke across three countries [file sj-docx-1-wso-10.1177_17474930231203149.docx]

**Supplementary**

***Data Source***

We used the UK, Indian and Qatar arms of the ongoing large prospective international Bio-Repository of DNA in Stroke (BRAINS) study. This hospital-based study meets all ethical standards set by local Institutional Review Boards and has received full institutional ethics approval in the participating countries. UK stroke patients were screened at 21 participating hospital sites located within London, Sussex, Surrey, West Yorkshire, West Midlands, Kent, Bedfordshire, and Lancashire. The recruitment period was between 2010 and 2019. The sites were chosen to include regions with high South Asian populations while also being representative of the white British population. The Indian arm screened stroke patients at two hospital sites located in New Delhi (All India Institute of Medical Sciences, AIIMS) in northern India, and Kerala (Sree Chitra Tirunal Institute for Medical Sciences and Technology, SCTIMST) in southern India. The Qatar arm screened stroke patients at one hospital site located in its capital Doha (Hamad Medical Corporation, HMC). All cases were reviewed by a pre-designated onsite neurologist/stroke physicians with the diagnosis of ischemic stroke confirmed with computed tomography (CT) or magnetic resonance (MR) imaging. Stroke cases were subtyped using the TOAST (trial of ORG 10172 in acute stroke treatment) classification. Written informed consent was sought for each index case. For those unable to provide written consent, surrogate consent was taken. Extensive demographic data including age, sex, and ethnicity was collected during a nurse-led interview. All participants over 18 years of age at the time of stroke event were considered for the study to ensure a representative sample. Ethnicity was obtained via self-identification as Indian, Pakistani, Sri-Lankan, or Bangladeshi origin. Ethnicity was classified as South Asians living in India (ISA), South Asians living in Qatar (QSA), South Asians living in Britain UK (BSA), and white British UK stroke (WB) patients. White British and white Irish was classified as white British. All analyses in this study compared ISA, QSA and BSA with WB patients as the reference group.

Risk factors for cases were defined as: hypertension diagnosed at discharge (≥140/90mmHg), previous diagnosis of hypertension or pre-stroke treatment with antihypertensive; hypercholesterolemia defined by previous diagnosis or serum cholesterol >5.2 mmol/L; diabetes mellitus classified from a previous diagnosis of type I or II. Previous diagnosis of ischemic heart disease and atrial fibrillation data was collected from clinical records. Smoking and alcohol history were recorded if the patient uses/used on a regular basis. Central obesity is classified by increased waist circumference (men: >102cm, women: >88cm) or BMI (≥27) ^1^.

***Stakeholder involvement***

A not-for-profit stroke patient support group advised on the original protocol for recruitment of patients. Appropriate ethical approval for this study was obtained in the UK, India, and Qatar.

**References**

^1^ Han TS, Lean ME. A clinical perspective of obesity, metabolic syndrome and cardiovascular disease. *JRSM Cardiovasc Dis* 2016; **5**: 2048004016633371.
